# Melanocyte loss dominates the vitiligo transcriptome: a rank-based meta-analysis of six independent studies

**DOI:** 10.64898/2026.02.07.26345817

**Published:** 2026-02-09

**Authors:** Xijin Ge

**Affiliations:** 1 Department of Mathematics and Statistics, South Dakota State University, Brookings, SD 57007, USA; 2 Orditus LLC, 2301 Research Park Way, Suite 240, Brookings, SD 57007, USA

**Keywords:** depigmentation, gene expression profiling, melanocyte markers, robust rank aggregation, single-cell RNA-seq

## Abstract

Vitiligo is an autoimmune disorder characterized by melanocyte destruction. We performed a rank-based meta-analysis of six independent transcriptomic studies (115 samples) spanning microarray, bulk and single-cell RNA-seq platforms to identify consensus signatures of lesional skin.

Robust Rank Aggregation identified 114 differentially expressed genes (FDR < 0.05) with striking asymmetry: 108 downregulated versus 6 upregulated. Downregulated genes were dominated by melanocyte markers (MLANA, TYRP1, DCT, PMEL, KIT). Upregulated genes included interferon-stimulated genes (OAS1, OAS2, EPSTI1). Pathway-level meta-analysis confirmed uniform suppression of melanogenesis, while immune activation was heterogeneous across datasets.

Single-cell data from three included studies confirmed melanocyte depletion. The 108 downregulated genes showed exclusive expression in melanocytes. These include neural genes (PLP1, GPM6B, NRXN3), consistent with melanocytes’ neural crest origin. We also identified candidate melanocyte markers such as CYB561A3 and QPCT with high melanocyte specificity and consistent downregulation in vitiligo.

These findings reveal a robust melanocyte loss signature in vitiligo detectable across all platforms, and study-dependent immune activation possibly influenced by sampling method and disease characteristics.

## Introduction

Vitiligo is an acquired depigmenting disorder affecting 0.5–2% of the global population (Ezzedine et al., 2015), characterized by the selective loss of melanocytes (Frisoli et al., 2020). Active depigmentation involves IFN-gamma signaling and the CXCL9/10-CXCR3 chemokine axis, which recruit autoreactive CD8+ T cells to the skin (Harris et al., 2012; Rashighi et al., 2014). In stable lesions, tissue-resident memory CD8+ T cells persist and can be reactivated, preventing stable repigmentation (Boniface et al., 2018; Richmond et al., 2018).

Genome-wide transcriptomic profiling has broadened this picture, revealing not only interferon-gamma-induced gene expression and immune cell infiltration (Rashighi et al., 2014; Xu et al., 2022) but also melanocyte-intrinsic changes and WNT pathway dysregulation (Regazzetti et al., 2015) and keratinocyte remodeling (Singh et al., 2017). However, the relative prominence of these signatures varies across studies, possibly reflecting biological heterogeneity (disease stage, patient population), technical factors (platform, sampling method), or limited sample sizes (typically 5–15 patients). A previous rank-based meta-analysis of three microarray datasets identified candidate biomarkers (Zhang et al., 2021), but a broader comparison across platforms is needed to distinguish robust consensus signatures from study-specific signals.

Here we performed meta-analysis of six independent vitiligo transcriptomic studies (115 samples; Table 1) spanning bulk RNA-seq, microarray, and single-cell pseudobulk platforms, after excluding one outlier dataset identified through leave-one-out validation. Using Robust Rank Aggregation (Kolde et al., 2012), we identified consensus differentially expressed genes without requiring effect size standardization, characterized dysregulation at both gene and pathway levels, and leveraged single-cell RNA-seq data from three studies (Shiu et al., 2022; Brunner et al., 2025; Xu et al., 2022) to validate signatures at the cell type level. Our findings reveal that melanocyte loss is robustly detected across platforms while immune activation is study-dependent.

## Results

### Study characteristics and data harmonization

**Figure 1.**
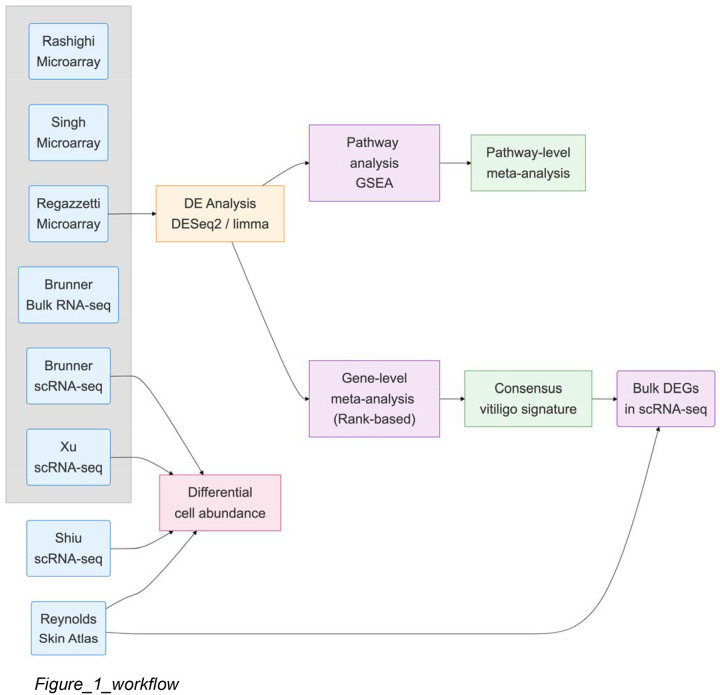
Study Overview and Meta-Analysis Workflow **Title:** Rank-based meta-analysis of vitiligo transcriptomic studies **Legend:** Schematic overview of the meta-analysis workflow. Differential expression results from six independent studies (Table 1) were harmonized to a common format, genes were ranked by test statistic within each study, and Robust Rank Aggregation (RRA) was applied to identify consensus differentially expressed genes. Leave-one-out analysis was performed to assess robustness, followed by pathway enrichment on the consensus signature.

We assembled transcriptomic data from seven independent vitiligo datasets encompassing 125 samples across bulk RNA-seq, microarray, and scRNA-seq (single cell RNA-Seq) platforms (Table 1, Figure 1). Five datasets employed paired designs comparing lesional versus non-lesional skin within patients, while two used unpaired designs comparing vitiligo patients to healthy controls.

Each dataset was uniformly reprocessed from raw data using DESeq2 (Love et al., 2014; RNA-seq) or limma (Ritchie et al., 2015; microarray), with stringent filtering of lowly expressed genes (Methods). For Brunner bulk RNA-seq, we used the differential expression results from the original publication (Brunner et al., 2025). Results were harmonized to a common schema of gene symbols, log2 fold changes (FC), test statistics, and false discovery rates (FDRs), with 8,064 genes detected across all seven studies.

### Limited overlap in differentially expressed genes (DEGs)

Exploratory analysis revealed substantial heterogeneity across studies. Statistical power varied considerably: studies with larger sample sizes showed strong enrichment for small p-values, while smaller studies exhibited near-uniform p-value distributions (Figure S1). Using conventional thresholds (FDR < 0.05, FC > 1.5), DEG counts ranged from 0 (Rashighi) to 3,154 (Singh), with limited overlap across studies, especially on upregulated genes (Table 1, Figures S2–S3). Pairwise log FC correlations were weak (−0.14 to 0.39; Figure S4), yet top-ranked genes showed consistent positioning across studies despite divergent DEG counts (Figures S5–S6). This pattern—poor agreement in effect sizes but concordant ranks—motivated rank-based rather than effect-size-based meta-analysis.

### Rank-based meta-analysis reveals direction-dependent robustness

For meta-analysis, genes were ranked by test statistic within each study, and Robust Rank Aggregation (RRA; Kolde et al., 2012) was used to identify genes ranking consistently higher (or lower) than expected by chance. RRA applied to 13,521 genes present in at least four of seven studies identified 222 differentially expressed genes at FDR < 0.05: 140 downregulated genes dominated by melanocyte markers and 82 upregulated genes enriched for interferon-stimulated genes.

Leave-one-out (LOO) analysis revealed direction-dependent stability (Figure S7). Downregulated genes were more robust (mean Jaccard = 0.40) than upregulated genes (mean Jaccard = 0.29). Removing the Shiu study caused dramatic loss of upregulated genes (Jaccard = 0.07; only 6 of 82 retained), while downregulated genes remained intact (Jaccard = 0.58).

Investigation revealed that Shiu-specific upregulated genes were dominated by MHC class II molecules and T cell markers, likely reflecting suction blister sampling, which captures epidermal immune cells more efficiently than conventional biopsies. To obtain a consensus signature comparable across platforms, we present results excluding the Shiu dataset, while acknowledging that this may underestimate immune activation in active disease.

### Downregulation of pigmentation genes is consistent

**Figure 2.**
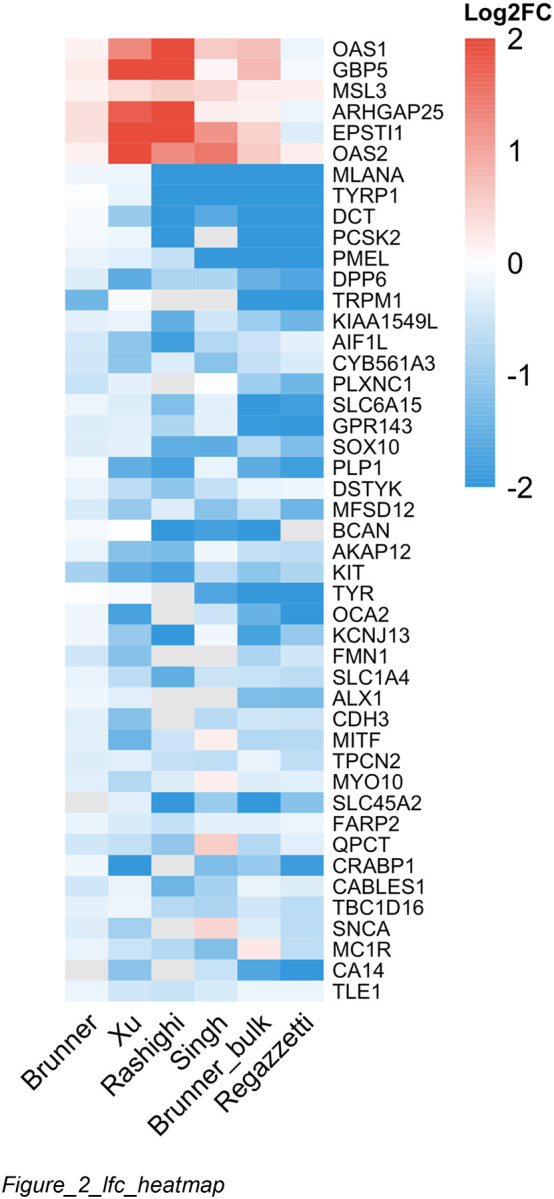
Consensus Differentially Expressed Genes **Title:** Cross-study log2 fold change heatmap for meta-analysis significant genes **Legend:** Heatmap of log2 fold changes for significant genes from the 6-study analysis (excluding Shiu) across all six studies. Melanocyte markers (top cluster, blue) show consistent strong downregulation; the few robust upregulated genes (bottom, red) are interferon-stimulated genes.

After excluding Shiu, RRA applied to the remaining six studies identified 114 differentially expressed genes at FDR < 0.05 (Figure 2): 108 downregulated (95%) and only 6 upregulated (5%), revealing a fundamental asymmetry in the vitiligo transcriptome.

The top downregulated genes include canonical melanocyte markers—MLANA, TYRP1, and DCT ranked first through third, followed by PMEL, TRPM1, KIT, and TYR (Figure 2). These genes showed 100% direction concordance across all studies that detected them, with mean log2FC ranging from −1.6 to −2.7. Additional melanocyte genes (SOX10, MITF, OCA2, SLC45A2, GPR143) were also significantly downregulated, collectively demonstrating profound melanocyte depletion (Figure S8). Unexpectedly, downregulated genes were also enriched for neural terms including synaptic transmission and axon development, a finding explored further in pathway analysis.

In contrast, only six genes achieved significance in the upregulated direction: OAS1, OAS2, EPSTI1, GBP5, MSL3, and ARHGAP25 (Figure 2). These include interferon-stimulated genes (OAS1, OAS2, EPSTI1, GBP5), consistent with the established role of type I/II interferon signaling in vitiligo (Harris et al., 2012; Frisoli et al., 2020). The small number of robust upregulated genes may reflect variable immune cell capture across tissue extraction methods and weaker underlying signal from infiltrating immune cells compared to the near-complete loss of melanocyte transcripts.

### Pathway analysis reveals downregulation of neural genes

**Figure 3.**
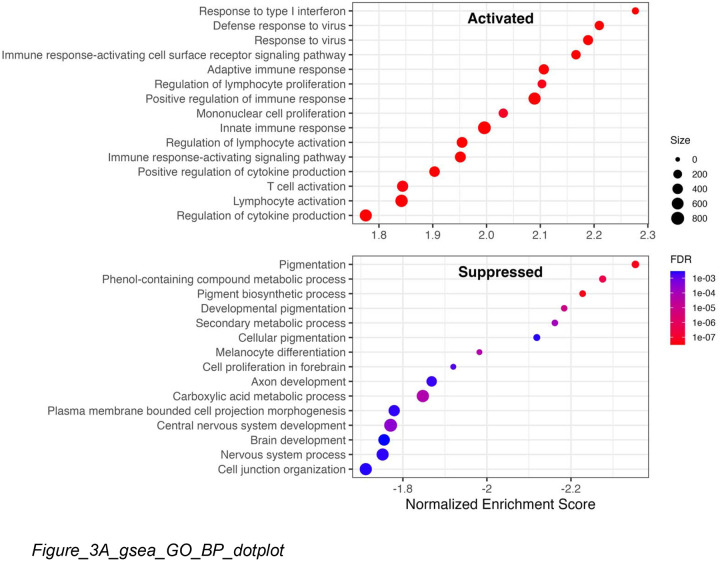

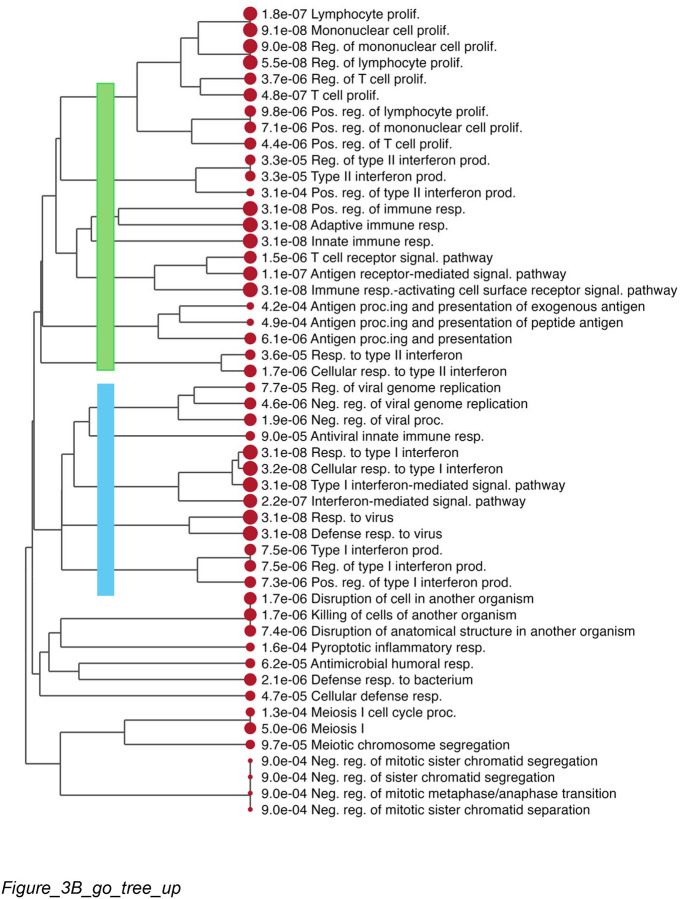

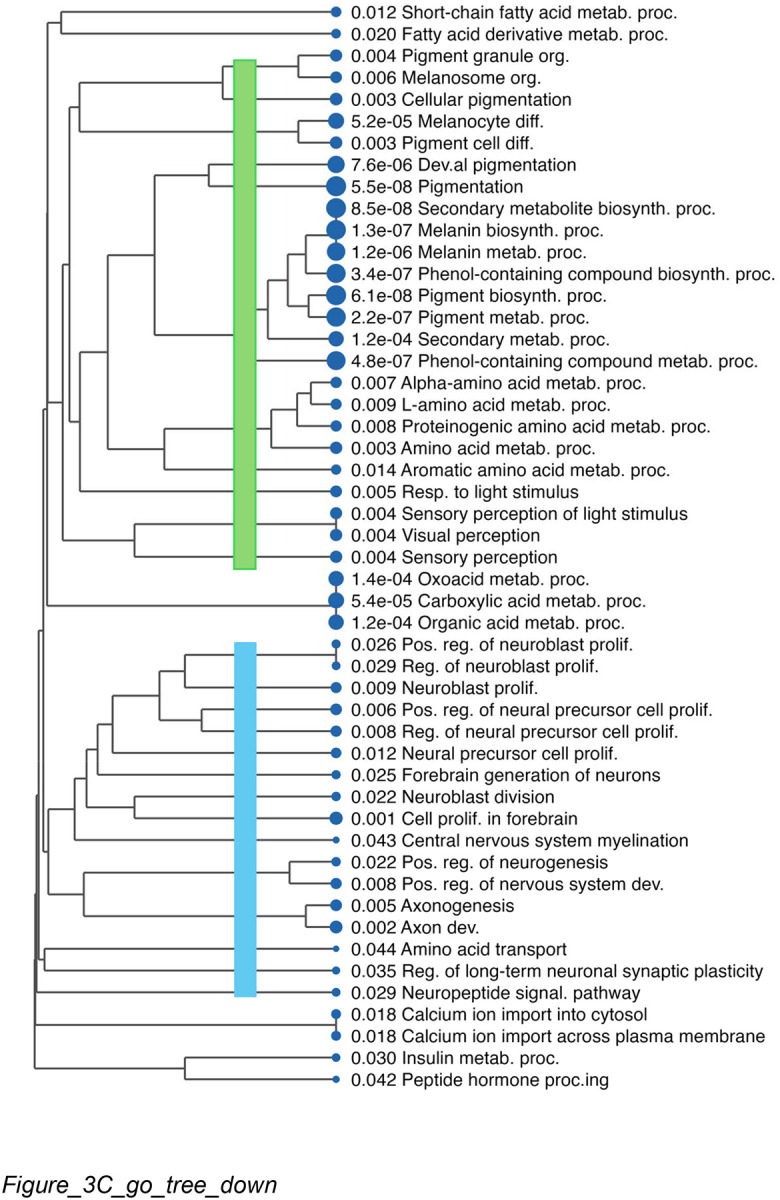
Pathway Enrichment Analysis of Consensus Signature **Title:** GSEA reveals activation of interferon response and suppression of melanogenesis **Legend:** (A) Combined dotplot of Gene Set Enrichment Analysis (GSEA) results for GO Biological Process terms. Top panel shows activated pathways (positive NES); bottom panel shows suppressed pathways (negative NES). Dot size indicates gene set size; color indicates FDR. Type I interferon signaling and antiviral defense pathways are strongly activated (NES > 2.0), while pigmentation and melanin biosynthesis pathways are profoundly suppressed (NES < −2.0). (B) Hierarchical tree visualization of activated GO terms clustered by Jaccard similarity of gene membership. Upregulated terms cluster into immune response, cell cycle progression, and ribosome biogenesis modules. (C) Hierarchical tree visualization of suppressed GO terms. Downregulated terms cluster into pigmentation, melanin biosynthesis, and neural development modules.

To identify dysregulated pathways, we performed GSEA using all genes ranked by pi-value (log FC × −log (p); Xiao et al., 2014), which incorporates both effect size and statistical significance while encoding direction. Top significantly enriched GO Biological Process terms are shown in Figure 3A. Activated pathways were dominated by type I interferon signaling and defense response to virus; suppressed pathways by pigmentation, melanin biosynthesis, and, to a lesser extent, nervous system-related processes.

Hierarchical clustering of the top 50 enriched terms by gene overlap revealed distinct functional modules (Figure 3B–C). Activated terms clustered into type I and type II interferon pathways. Suppressed terms included a large melanocyte function cluster (pigmentation, melanin biosynthesis, melanosome organization) and, notably, a separate cluster of neural development pathways—axon development, neuron projection morphogenesis, and central nervous system development (Figure 3C). This unexpected finding could represent noise if melanin pathway genes have dual functions. However, leading edge analysis confirmed minimal overlap: only 6 of 157 neural pathway genes (3.8%) were shared with melanocyte pathways. The neural-specific genes—including PLP1, NRXN3, and PLXNC1—showed significant downregulation (median log FC = −0.22, Wilcoxon p = 8 × 10 ^2^) with 80% cross-study consistency, suggesting the signal reflects genuine biology, albeit with smaller effect sizes than melanocyte genes. Because melanocytes derive from neural crest progenitors, this signal can also be driven by melanocyte loss rather than independent neural involvement.

### Pathway-level meta-analysis confirms cross-study concordance

**Figure 4.**
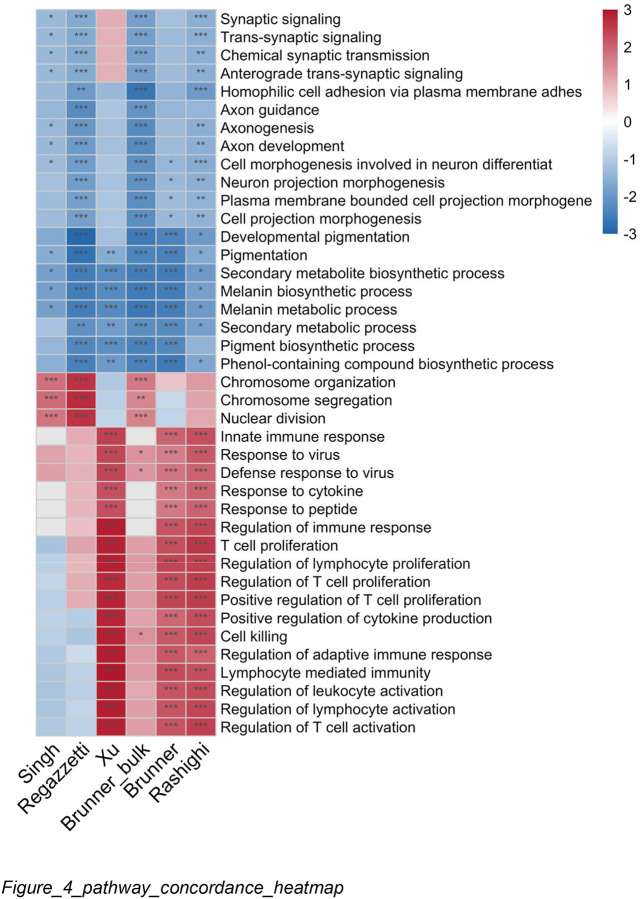
Pathway-Level Meta-Analysis Confirms Cross-Study Concordance **Title:** Per-study GSEA reveals concordant pathway dysregulation **Legend:** NES heatmap for top 40 GO Biological Process pathways (20 activated, 20 suppressed) across six studies. Color indicates NES (red = activated, blue = suppressed); asterisks indicate significance (*FDR < 0.05*, ***FDR < 0.01*,** FDR < 0.001). Melanogenesis and pigmentation pathways show consistent suppression across all studies (mean NES = −2.1). Immune pathways show activation in most studies but with greater heterogeneity.

To complement the gene-level meta-analysis, we performed GSEA on each study individually using test statistic rankings. Of 5,126 GO terms tested, 681 showed concordant enrichment (present in ≥4 studies, ≤1 discordant direction, ≥2 significant at FDR < 0.05). Per-study normalized enrichment scores (NES) revealed consistent patterns (Figure 4): pigmentation pathways showed uniform suppression across all six studies, while immune pathways showed more variable concordance.

Per-study results were combined using Stouffer’s signed Z-method (Whitlock, 2005), where discordant effects cancel rather than accumulate significance. The top suppressed pathways were pigmentation (FDR = 4.9 × 10 ^2^) and melanin metabolism (FDR = 1.0 × 10 ^21^); the top activated pathways were innate immune response (FDR = 1.7 × 10 ^1^) and T cell proliferation (FDR = 3.3 × 10 ^1^).

This analysis confirmed the unexpected suppression of neural development pathways. Multiple neural pathways—axon development, axonogenesis, neuron projection morphogenesis—ranked among the most robustly suppressed, with effect sizes comparable to pigmentation. Critically, all six datasets showed independent suppression, with four to six reaching individual significance. Leading edge analysis identified core contributors including melanocyte markers (SOX10, DCT) alongside neural-associated genes (NGFR, L1CAM, PLP1, NRXN3, GPM6B), the last of which reached gene-level significance in the meta-analysis (FDR = 0.017).

### Melanocyte loss explains most downregulated genes

**Figure 5.**
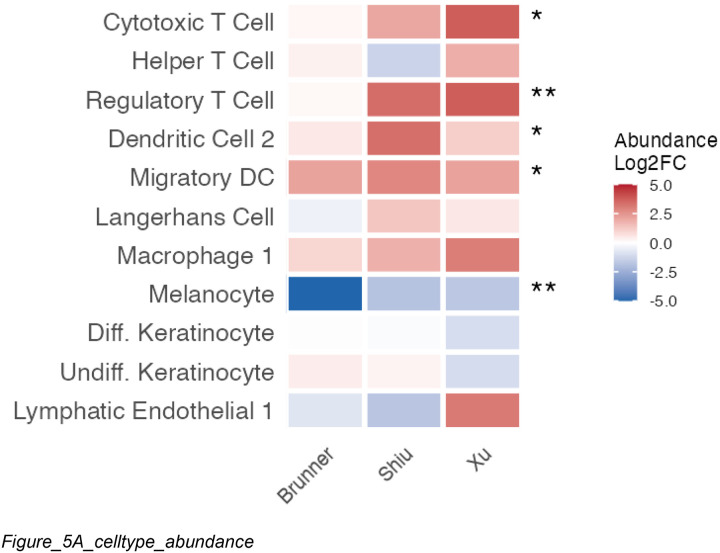

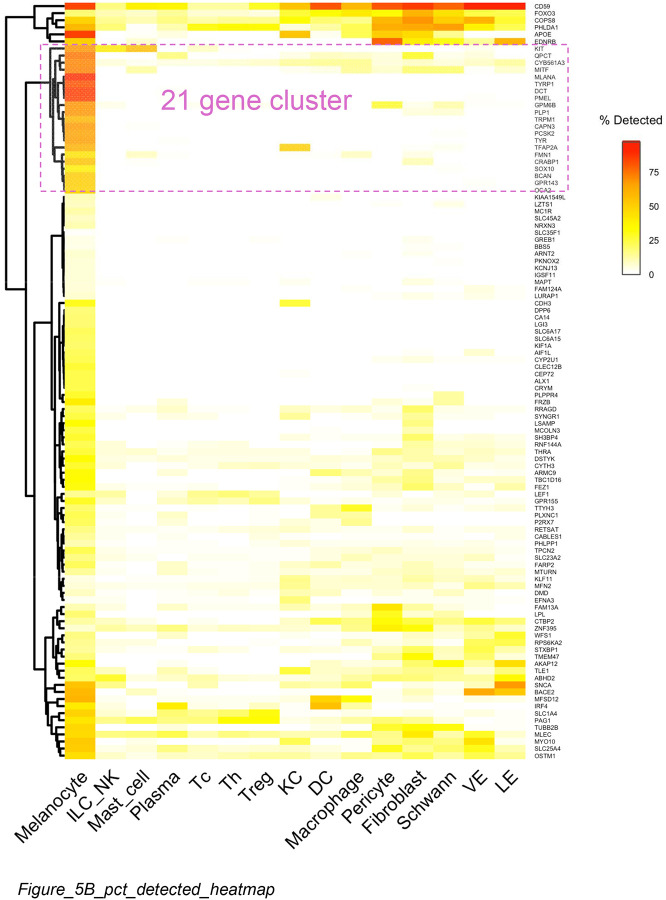
Cell Type Composition Changes Validate Transcriptomic Findings **Title:** Single-cell RNA-seq validates melanocyte loss and localizes consensus signature to melanocytes **Legend:** (A) Heatmap showing log2 fold change in cell type abundance between lesional and control skin across three scRNA-seq studies (Brunner, Shiu, Xu). Cell types were annotated by label transfer from the Reynolds healthy skin atlas. Proportions were compared using propeller (unpaired design) or limma with patient blocking (paired designs). Asterisks indicate significance within individual studies (*FDR < 0.1, **FDR < 0.05). Melanocytes show consistent depletion across all three studies (mean log2FC = −2.87), validating the bulk transcriptomic findings at the cellular level. T cell populations and dendritic cells show enrichment consistent with immune infiltration, though with greater heterogeneity across studies. (B) Percent detection heatmap for meta-analysis downregulated genes (RRA FDR < 0.05) across cell types. Hierarchical clustering of genes by detection pattern reveals a melanocyte-specific cluster containing canonical pigmentation genes (TYRP1, DCT, MLANA, PMEL, TYR). These genes are detected in >75% of melanocytes but <25% of other cell types, confirming that the consensus downregulated signature is driven by melanocyte-specific transcripts rather than broadly expressed genes.

To investigate whether transcriptomic changes reflect altered cell type composition, we analyzed cell type abundance from three scRNA-seq datasets (Brunner, Shiu, Xu). Cells were annotated using reference-based label transfer from the Reynolds healthy skin atlas (Reynolds et al., 2021), and abundance changes between lesional and normal skin were tested (Figure 5A). Per-study p-values were combined using Stouffer’s signed Z-method (Whitlock, 2005).

As expected, melanocytes showed consistent depletion across all three studies (mean log2FC = −2.87; combined FDR = 0.003), aligning with the transcriptomic signature dominated by downregulated melanocyte markers. Several immune cell types were significantly enriched, including Tregs (FDR = 0.007), cytotoxic T cells (FDR = 0.015), migratory dendritic cells (FDR = 0.015), and DC2 (FDR = 0.030), confirming the dual hallmarks of vitiligo: melanocyte loss accompanied by infiltration of antigen-presenting and cytotoxic immune cells.

To test whether melanocyte loss fully explains the downregulated signature, we examined cell type-specificity of the 108 downregulated genes using percent detection across cell types in the healthy skin atlas (Figure 5B). The vast majority were detected far more frequently in melanocytes than other cell types. This is confirmed by average expression analysis (Figure S10). Our analysis provides strong evidence that melanocyte loss drives the downregulated transcriptomic signature of vitiligo.

### Downregulated genes include melanocyte markers

The heatmap of the detection rate of downregulated genes across cell types (Figure 5B) includes a distinct cluster of 21 genes with near-exclusive melanocyte expression (Table S2). This cluster included established pigmentation genes essential for melanin synthesis (TYR, TYRP1, DCT), melanosome structure (PMEL, TRPM1), and melanocyte transcription (MITF, SOX10, TFAP2A) (Kenny et al., 2022). Additional melanocyte-associated genes included the receptor tyrosine kinase KIT, melanosomal membrane proteins OCA2 and GPR143 (mutations in which cause oculocutaneous and ocular albinism, respectively), and the retinoic acid-binding protein CRABP1 (Collins and Watt, 2008). This 21-gene cluster, defined by near-exclusive melanocyte expression and consistent downregulation in vitiligo, is enriched for established melanocyte markers.

Several genes in this cluster have documented neural functions yet showed striking melanocyte specificity over Schwann cells—another neural crest derivative in skin. BCAN (brevican, detected in 49.5% of melanocytes vs. 0.4% of Schwann cells), PCSK2 (63% vs. 1.1%), and PLP1 (66% vs. 6.7%) all showed >10-fold enrichment in melanocytes despite their canonical neural associations.

The cluster also contained less-characterized genes with emerging evidence of melanocyte expression. CYB561A3, a lysosomal ferrireductase recently identified as a pigmentation regulator through GWAS in African populations (Feng et al., 2024), was detected in 69% of melanocytes versus 8% of other cell types. QPCT (glutaminyl cyclase) showed 72% detection in melanocytes versus <10% in other cell types, a pattern validated across four independent datasets including the Reynolds atlas and three vitiligo scRNA-seq studies. QPCT protein has been detected in hyperpigmented skin (Yin et al., 2015) and is highly expressed in melanoma (Gillis, 2006), yet its function in normal melanocyte biology remains unexplored. These and other genes in the 21-gene cluster warrant functional investigation in the context of vitiligo.

## Discussion

The striking asymmetry of the vitiligo transcriptome—108 downregulated versus 6 upregulated genes—likely reflects a difference in signal characteristics. Melanocyte loss produces large, consistent transcriptomic changes, while immune-related changes appear weaker and diverse.

Several factors may explain why immune signals are difficult to capture. Small sample sizes (5–15 patients) limit power for variable signals. In paired designs, non-lesional skin is not immunologically quiescent—resident memory T cells and subclinical immune networks persist in clinically normal-appearing skin (Gellatly et al., 2021; Migayron et al., 2025)—compressing the lesional-versus-non-lesional differential. Uncontrolled variation in disease activity (Speeckaert et al., 2023) and anatomical site (Marella et al., 2025) further obscure immune signals. These observations do not diminish the established role of CXCR3 chemokine signaling in vitiligo (Speeckaert et al., 2023), but suggest that bulk transcriptomics with small cohorts may be insufficient to reliably capture immune signatures in this disease.

Our meta-analysis also identified a consistent suppression of neural development pathways. Re-analysis of single-cell datasets indicates that this signal can be largely explained by melanocyte expression profiles: genes driving the enrichment—PLP1, GPM6B, BCAN, PCSK2—were detected in >50% of melanocytes but <7% of Schwann cells (Figure 5B), consistent with melanocytes’ neural crest origin (Adameyko et al., 2009; Brombin and Patton, 2024). The roles of these genes in melanocyte biology have not been characterized and warrant investigation. More broadly, this result illustrates that when cell types sharing developmental origins are present in the same tissue, pathway enrichment may reflect changes in cell composition rather than pathway dysregulation.

The 21-gene melanocyte cluster (Table S1), downregulated in vitiligo and exclusively detected in melanocytes, includes established pigmentation genes alongside less-characterized candidates. CYB561A3, a lysosomal ferrireductase linked to pigmentation through GWAS in African populations (Feng et al., 2024), was detected in 69% of melanocytes versus 8% of other cell types. QPCT (glutaminyl cyclase, 72% melanocyte detection) has been detected at the protein level in hyperpigmented skin (Yin et al., 2015) and is highly expressed in melanoma (Gillis, 2006); recent work showing that QPCT pyroglutamates the melanocyte surface protein TYRP1 (van der Plas-van Duijn et al., 2025) suggests a direct role in pigmentation biology. The consistency of melanocyte marker detection across all platforms and study designs in this meta-analysis suggests these genes may have utility as biomarkers, in contrast to immune markers whose detection varied with sampling method and study design.

This study has several limitations. The low cross-study effect size correlation (mean r = 0.11) justified rank-based meta-analysis, but this approach cannot provide pooled effect size estimates or distinguish weak consistent signals from strong signals in few studies. The heterogeneous comparisons—lesional versus non-lesional in paired studies, vitiligo versus healthy controls in unpaired studies—may capture different aspects of pathology. Platform differences, including pseudobulk aggregation and FFPE tissue, may introduce biases not fully captured by ranking. Most studies did not characterize disease activity, limiting phenotypic stratification.

In summary, meta-analysis across six heterogeneous transcriptomic studies reveals that the downregulated genes are almost entirely melanocyte-derived, including neural crest-associated genes whose expression in melanocytes has been underappreciated. Immune signatures, though detectable at the pathway level, are weak and variable at the gene level, likely reflecting differences in sampling method, disease activity, and study design. Integrating bulk meta-analysis with single-cell data proved essential for interpreting these signals and identified candidate melanocyte biomarkers—including CYB561A3, QPCT, and PCSK2—that warrant functional characterization. Future studies with larger, well-characterized cohorts and standardized sampling are needed to resolve the immune component of the vitiligo transcriptome.

## Materials and Methods

### Study Selection and Data Sources

We searched GEO and published literature for transcriptomic studies comparing vitiligo-affected skin to unaffected skin. Inclusion criteria were: (1) human samples, (2) genome-wide expression profiling (microarray or RNA-seq), (3) comparison of lesional versus non-lesional or vitiligo versus healthy control skin, and (4) availability of raw or processed expression data. Seven studies were initially included; one (Shiu) was subsequently excluded based on leave-one-out validation, yielding six studies comprising 115 samples (Table 1). Data were obtained from GEO (GSE298871, GSE75819, GSE65127, GSE53146, GSE203262) and the China National Center for Bioinformation (OMIX691). Platforms spanned Illumina BeadChip, Affymetrix microarray, bulk RNA-seq, and pseudobulk from single-cell RNA-seq.

### Individual Study Re-Analysis

Five paired studies compared lesional to non-lesional skin within the same patients: Singh (n = 30, Illumina BeadChip, limma), Regazzetti (n = 20 from 10 pairs, Affymetrix, limma), Brunner bulk RNA-seq (n = 30, published DE results), Brunner pseudobulk (n = 10, DESeq2), and Shiu pseudobulk (n = 10 from 5 pairs after 1 patient excluded, DESeq2). Two unpaired studies compared vitiligo to control skin: Rashighi (n = 10, Illumina DASL for FFPE tissue, limma; 1 outlier excluded, 9 analyzed) and Xu (n = 15, pseudobulk DESeq2). For scRNA-seq datasets, pseudobulk profiles were generated by aggregating counts across all QC-passed cells within each sample. Microarray data were processed from raw files with standard normalization (quantile for Illumina, RMA for Affymetrix). For Brunner bulk RNA-seq, log2 fold changes and FDR-adjusted p-values were obtained from the original publication, as raw data were not available; test statistics were recovered from p-values using the inverse normal cumulative distribution function. Detailed processing for each study is described in Supplementary Methods.

### Data Harmonization and Meta-Analysis

Differential expression results were harmonized to a common schema: gene symbol, log2 fold change, standard error, test statistic (t-statistic for limma, Wald statistic for DESeq2), and p-values. Traditional inverse-variance meta-analysis was not appropriate due to standard error miscalibration across platforms and low cross-study effect size correlations (mean Pearson r = 0.11). We therefore applied Robust Rank Aggregation (RRA), a non-parametric method that identifies genes ranked consistently highly across studies without requiring effect size standardization (Kolde et al., 2012). Genes were ranked within each study by absolute test statistic, and RRA was run separately for upregulated and downregulated genes; in each direction, genes with concordant fold change were placed at the top of the ranked list while genes with discordant fold change were appended at the bottom, penalizing inconsistent directions. Only genes present in at least four studies were included. P-values were corrected using the Benjamini-Hochberg method.

### Robustness Analysis

Leave-one-out analysis assessed stability by removing each study in turn and computing Jaccard similarity between the resulting gene set and the full analysis. The Shiu study was excluded from the final meta-analysis based on low Jaccard similarity (0.073 for upregulated genes), indicating it disproportionately influenced the results.

### Pathway Enrichment Analysis

Gene Set Enrichment Analysis (GSEA) used the full ranked gene list (ordered by pi-value = median log2FC × −log10(RRA p-value); Xiao et al., 2014) to test Gene Ontology Biological Process and KEGG pathway enrichment using clusterProfiler (Wu et al., 2021; gene set size 15–800). Over-representation analysis (ORA) tested meta-analysis significant genes (FDR < 0.05) against the same databases (gene set size 10–500). GO terms were clustered by semantic similarity using simplifyEnrichment (Gu and Hübschmann, 2023). To assess whether suppressed neural development pathways reflected genuine biology or annotation overlap with melanocyte genes (shared neural crest origin), we performed leading edge analysis. Genes from GSEA core enrichment were extracted for neural-related pathways (identified by keywords: neuro, axon, neural, nerve, synap, glia, brain, nervous) and melanocyte pathways (melano, pigment, melanin, tyrosin), and overlap was quantified using Jaccard similarity and a hypergeometric test. Effect sizes were compared across gene categories using Kruskal-Wallis and pairwise Wilcoxon tests.

### Pathway-Level Meta-Analysis

To assess pathway concordance across studies, GSEA was performed on each study individually. Per-study normalized enrichment scores were combined using Stouffer’s Z-method with signed Z-scores (Whitlock, 2005), ensuring discordant directions cancel rather than accumulate significance. Pathways were considered concordant if present in ≥4 studies with ≤1 discordant direction and significant in ≥2 studies.

### Cell Type Proportion Analysis from scRNA-seq data

To assess whether bulk transcriptomic signatures reflected changes in cell type composition, we analyzed three scRNA-seq datasets (Xu, Brunner, Shiu; Shiu was included in cell composition analysis despite exclusion from the gene-level meta-analysis). Cell types were annotated by reference-based label transfer from the Reynolds healthy skin atlas (Reynolds et al., 2021) using Seurat v5, with annotation confidence assessed by prediction score and entropy filtering. Annotations were validated against canonical markers. Cell type proportions were compared between conditions using limma (paired studies) or propeller (Phipson et al., 2022; unpaired), with arcsin-square root transformation for variance stabilization. Cross-study evidence was combined using Stouffer’s Z-method. Details are provided in Supplementary Methods.

### Cell Type Specificity of Meta-Analysis Genes

To assess whether the downregulated meta-analysis signature reflects melanocyte loss, we examined cell type specificity of the 108 downregulated genes using percent detection (percentage of cells with non-zero expression) and average expression across cell types from the Reynolds healthy skin atlas. Genes were hierarchically clustered (Ward’s D2 method), revealing a 21-gene cluster with near-exclusive melanocyte expression (Table S1).

### Statistical Analysis

All analyses were performed in R (v4.5.2). Multiple testing correction used the Benjamini-Hochberg method with FDR < 0.05 considered significant. Key packages: limma (v3.66.0; Ritchie et al., 2015), DESeq2 (v1.48.0; Love et al., 2014), RobustRankAggreg (v1.2.1; Kolde et al., 2012), clusterProfiler (v4.18.4; Wu et al., 2021), fgsea (v1.36.2; Korotkevich et al., 2021), simplifyEnrichment (v1.10.0; Gu and Hübschmann, 2023), Seurat (v5.4.0; Hao et al., 2024), and speckle (v1.4.0; Phipson et al., 2022).

## Supplementary Material

Supplement 1 Figure S1. Distribution of Differential Expression Statistics
**Title:** P-value and log FC distributions by study
**Legend:** P-value distributions (top) and log fold change distributions (bottom) for each study. Studies with larger sample sizes (Singh, Brunner bulk) show enrichment for small p-values, indicating stronger statistical power. Brunner pseudobulk and Rashighi show nearly uniform p-value distributions reflecting limited power. Log fold change distributions are centered near zero with study-specific variance; Brunner pseudobulk has the narrowest distribution, reflecting conservative effect size estimates from the small sample.
Figure S2. Gene Overlap Across Studies (Upregulated)
**Title:** UpSet plot of upregulated genes
**Legend:** UpSet plot showing intersection sizes for upregulated genes (FDR < 0.05, log FC > 0.58) across studies. Limited overlap is observed, with most genes detected in only one or two studies.
Figure S3. Gene Overlap Across Studies (Downregulated)
**Title:** UpSet plot of downregulated genes
**Legend:** UpSet plot showing intersection sizes for downregulated genes (FDR < 0.05, log FC < −0.58) across studies. Downregulated genes show slightly better overlap than upregulated genes, with melanocyte markers appearing across multiple studies.
Figure S4. Cross-Study Effect Size Correlation
**Title:** Pairwise log FC correlation matrix
**Legend:** Pairwise Pearson correlations of log fold changes for 11,988 genes present in at least 5 studies. Correlations are generally low (range: −0.14 to 0.39), indicating substantial heterogeneity across studies and platforms. The highest correlation is between Shiu and Xu (r = 0.39), both pseudobulk datasets. Low cross-study correlation justifies rank-based meta-analysis over traditional effect-size pooling methods.
Figure S5. Rank Agreement Across Studies (Downregulated)
**Title:** Study percentile ranks for top downregulated genes
**Legend:** Heatmap showing study-specific percentile ranks for top 50 downregulated genes ordered by median log FC. Lower percentile (orange/red) indicates higher rank within that study. Despite divergent DEG counts across studies (0 to 3,154), top-ranked downregulated genes show consistent ranking across platforms. Melanocyte markers (TYRP1, MLANA, KIT, DCT) rank in the top percentiles across nearly all studies, demonstrating that rank-based aggregation captures concordant signals missed by threshold-based approaches.
Figure S6. Rank Agreement Across Studies (Upregulated)
**Title:** Study percentile ranks for top upregulated genes
**Legend:** Heatmap showing study-specific percentile ranks for top 50 upregulated genes ordered by median log FC. Lower percentile (orange/red) indicates higher rank within that study. Upregulated genes show more heterogeneous ranking patterns compared to downregulated genes, reflecting variable immune cell capture across platforms.
Figure S7. Leave-One-Out Robustness Analysis
**Title:** Jaccard similarity by direction in leave-one-out analysis
**Legend:** Jaccard similarity between leave-one-out (LOO) gene sets and the full 7-study analysis, separated by direction. Downregulated genes show consistently higher stability (mean Jaccard = 0.40) than upregulated genes. Removing the Shiu study causes the most dramatic loss of upregulated genes (Jaccard = 0.073), while downregulated genes remain largely intact (Jaccard = 0.58).
Figure S8. Over-Representation Analysis
**Title:** ORA of differentially expressed genes
**Legend:** Over-representation analysis (ORA) dotplot showing fold enrichment for GO
Biological Process terms among upregulated (top) and downregulated (bottom) genes (FDR < 0.05 from RRA). Upregulated genes are enriched for interferon and antiviral response terms; downregulated genes are strongly enriched for pigmentation, melanin biosynthesis, and melanocyte development. Note: upregulated ORA results are driven by only 6 significant genes (OAS1, OAS2, GBP5, MSL3, ARHGAP25, EPSTI1).
Figure S9. Shiu Study Investigation
**Title:** Melanocyte marker comparison between Shiu and consensus
**Legend:** Comparison of melanocyte marker log FC between Shiu and 6-study mean.
While direction is concordant for most markers, effect sizes in Shiu are 3–5× smaller than the consensus, consistent with dilution of melanocyte signal by abundant immune cell transcripts in pseudobulk aggregation.
Figure S10. Average Expression of Downregulated Genes Across Cell Types
**Title:** Average expression heatmap confirms melanocyte specificity of downregulated genes
**Legend:** Mean log-normalized expression of meta-analysis downregulated genes (RRA FDR < 0.05) across cell types in the Reynolds healthy skin atlas. Hierarchical clustering reveals a melanocyte-specific cluster (bottom) containing canonical pigmentation genes (TYR, TYRP1, DCT, PMEL, MLANA) with expression levels 2–3 log units higher in melanocytes than other cell types. This complements the percent detection analysis (Figure 5B) by demonstrating that melanocyte-specific genes are not only more frequently detected but also expressed at substantially higher levels in melanocytes.

Supplement 2 Table S1 **Title:** Complete Meta-Analysis Results for All Genes
**Legend:** Full Robust Rank Aggregation (RRA) results for all 13,521 genes tested.
Columns include: gene symbol, RRA p-value, FDR-adjusted p-value, direction (UP or DOWN), number of studies detecting the gene, per-study rank percentiles, mean log2 fold change across studies, and direction consistency (proportion of studies with concordant direction).

Supplement 3 Table S2 **Title:** Melanocyte Marker Gene Cluster Expression Metrics
**Legend:** Expression characteristics of the 21-gene melanocyte marker cluster identified by hierarchical clustering of RRA-downregulated genes. Genes were clustered based on % detection across cell types in the Reynolds healthy skin single-cell atlas (~500,000 cells). Columns include: gene symbol, known melanocyte role, % cells with detected expression in melanocytes (% Det Mel), mean % detection across 14 non-melanocyte cell types (% Det Other), mean log-normalized expression in melanocytes (Expr Mel), and mean expression in other cell types (Expr Other). Established melanocyte markers (bold) include genes with well-characterized pigmentation functions; other genes represent potentially novel melanocyte-associated markers.

## Figures and Tables

**Table 1.** Studies Included in the Meta-Analysis

Study	Accession	Disease	Platform	Samples	Genes	DEGs^a^
Brunner 2025	GSE298871	NSV	scRNA-seq	5 pairs	16,133	2
Brunner 2025 (bulk)	Supp. Table	NSV	RNA-seq	15 pairs	19,773	1,344
Singh 2017	GSE75819	Stable NSV	Illumina	15 pairs	16,312	3,154
Regazzetti 2015	GSE65127	Active NSV	Affymetrix	10 pairs	13,908	141
Rashighi 2014	GSE53146	Active vitiligo (FFPE)	Illumina	5 V + 5 C	12,707	0
Xu 2021	OMIX691	NSV	scRNA-seq	10 V + 5 H	13,080	1,239
Shiu 2022^b^	GSE203262	Stable vitiligo (blister)	scRNA-seq	5 pairs	11,995	133

a Number of differentially expressed genes (FDR < 0.05 and |log FC| > 0.58 [FC > 1.5]).

b Excluded from the final meta-analysis based on leave-one-out validation.

c C, control; V, vitiligo; H, healthy; NSV, nonsegmental vitiligo; scRNA-seq, single-cell RNA sequencing; FFPE, formalin-fixed paraffin-embedded.

## Data Availability

This study is a secondary analysis of publicly available datasets. All source data are available from the following repositories: Gene Expression Omnibus (GEO): GSE298871 (Brunner pseudobulk), GSE75819 (Singh), GSE65127 (Regazzetti), GSE53146 (Rashighi), and GSE203262 (Shiu). China National Center for Bioinformation: OMIX691 (Xu). Brunner bulk RNA-seq differential expression results were obtained from Table S3 of the original publication (Brunner et al., 2025). Raw sequencing data for this cohort were not publicly available at the time of analysis. All analysis code is available at https://github.com/gexijin/vitiligo. The complete analytical workflow is tracked in the commit history. This repository also contains all prompts, slash commands, and custom Claude Skills used in this project. A Docker virtual machine image preserving the exact computing environment is available at https://hub.docker.com/repository/docker/gexijin/vitiligo/ to ensure reproducibility.

## Abbreviations

DE: differential expression
DEG: differentially expressed gene
FC: fold change
FDR: false discovery rate
FFPE: formalin-fixed paraffin-embedded
GEO: Gene Expression Omnibus
GO: Gene Ontology
GSEA: Gene Set Enrichment Analysis
GWAS: genome-wide association study
IFN: interferon
KEGG: Kyoto Encyclopedia of Genes and Genomes
LOO: leave-one-out
MHC: major histocompatibility complex
NES: normalized enrichment score
ORA: over-representation analysis
RMA: Robust Multi-array Average
RRA: Robust Rank Aggregation
scRNA-seq: single-cell RNA sequencing
